# Kalpra: A kernel approach for longitudinal pathway regression analysis integrating network information with an application to the longitudinal PsyCourse Study

**DOI:** 10.3389/fgene.2022.1015885

**Published:** 2022-12-06

**Authors:** Bernadette Wendel, Markus Heidenreich, Monika Budde, Maria Heilbronner, Mojtaba Oraki Kohshour, Sergi Papiol, Peter Falkai, Thomas G. Schulze, Urs Heilbronner, Heike Bickeböller

**Affiliations:** ^1^ Department of Genetic Epidemiology, University Medical Center Göttingen, Georg-August-University Göttingen, Göttingen, Germany; ^2^ Institute of Psychiatric Phenomics and Genomics (IPPG), University Hospital, LMU Munich, Munich, Germany; ^3^ Department of Psychiatry and Psychotherapy, University Hospital, LMU Munich, Munich, Germany; ^4^ Department of Psychiatry and Behavioral Sciences, SUNY Upstate Medical University, Syracuse, NY, United States; ^5^ Department of Psychiatry and Behavioral Sciences, Johns Hopkins University School of Medicine, Baltimore, MD, United States

**Keywords:** pathway analysis, kernel machine regression, longitudinal data, network, PsyCourse Study

## Abstract

A popular approach to reduce the high dimensionality resulting from genome-wide association studies is to analyze a whole pathway in a single test for association with a phenotype. Kernel machine regression (KMR) is a highly flexible pathway analysis approach. Initially, KMR was developed to analyze a simple phenotype with just one measurement per individual. Recently, however, the investigation into the influence of genomic factors in the development of disease-related phenotypes across time (trajectories) has gained in importance. Thus, novel statistical approaches for KMR analyzing longitudinal data, i.e. several measurements at specific time points per individual are required. For longitudinal pathway analysis, we extend KMR to long-KMR using the estimation equivalence of KMR and linear mixed models. We include additional random effects to correct for the dependence structure. Moreover, within long-KMR we created a topology-based pathway analysis by combining this approach with a kernel including network information of the pathway. Most importantly, long-KMR not only allows for the investigation of the main genetic effect adjusting for time dependencies within an individual, but it also allows to test for the association of the pathway with the longitudinal course of the phenotype in the form of testing the genetic time-interaction effect. The approach is implemented as an R package, *kalpra*. Our simulation study demonstrates that the power of long-KMR exceeded that of another KMR method previously developed to analyze longitudinal data, while maintaining (slightly conservatively) the type I error. The network kernel improved the performance of long-KMR compared to the linear kernel. Considering different pathway densities, the power of the network kernel decreased with increasing pathway density. We applied long-KMR to cognitive data on executive function (Trail Making Test, part B) from the PsyCourse Study and 17 candidate pathways selected from Reactome. We identified seven nominally significant pathways.

## 1 Introduction

Pathway analyses or gene-set analyses are association studies, which test whole gene sets or pathways for association with a phenotype of interest (Holmans, 2010; Mooney and Wilmot, 2015). In contrast to a genome-wide association analysis (GWAS) in which a great number of individual SNP association tests are performed, a smaller group of genes or SNPs is tested simultaneously. Thus, the multiple testing problem of a GWAS is tremendously mitigated. In the last two decades, many different general approaches and particular tools have been developed for pathway analysis (Holmans, 2010; Mooney and Wilmot, 2015; de Leeuw et al., 2016).

In this paper, we focus on kernel machine regression (KMR) (Liu et al., 2007; Ge et al., 2016), a machine-learning algorithm (Liu et al., 2007) with great flexibility. KMR is a semi-parametric regression analysis (Liu et al., 2007) initially designed to analyze case-control studies (Liu et al., 2008; Wu et al., 2010; Wu et al., 2011) or quantitative data (Liu et al., 2007; Wu et al., 2011; Ge et al., 2016). KMR models the environmental (non-genetic) parameters parametrically and the high-dimensional genetic data (e.g., genotype information) non-parametrically (Liu et al., 2007; Freytag et al., 2013). The genetic data are transformed into a similarity matrix containing for every pair of individuals quantitative values, which describe the genetic similarities of the pairs of individuals (Schaid, 2010; Ge et al., 2016). This matrix is denoted as kernel matrix. The transformation is performed by a kernel function, which can have different forms depending on the desired similarity concept (Freytag et al., 2013). There are many possibilities to model a pathway as the only requirement of the kernel function is to be positive semidefinite (Schaid, 2010; Schaid, 2010) For example, a popular kernel is the linear kernel (Wu et al., 2010; Freytag et al., 2013). New kernels have been also defined, e.g., a kernel adjusting for size bias (Freytag et al., 2012) or a kernel integrating the network information of a pathway (Freytag et al., 2013). The latter was possible thanks to the development of different pathway databases, e.g., Reactome (Jassal et al., 2019), Pathway Commons (Rodchenkov et al., 2019) or KEGG (Kanehisa et al., 2017). Different versions and extensions of KMR have been developed to address various research questions (for a summary see (Larson et al., 2019)). KMR analyzing more complex phenotype data, e.g., family samples (Malzahn et al., 2014; Yan et al., 2015) is just one example.

Longitudinal studies assess multiple, thus correlated, measurements over time for each single individual (Molenberghs and Verbeke, 2000; Caruana et al., 2015). They enable researchers to study the time course of the investigated phenotype. A number of statistical methods have been and are still being developed especially in this context. An important aspect of longitudinal studies is the frequently high number of missing data or unequal measurement points (Caruana et al., 2015). A popular method to overcome this challenge are linear mixed models (LMM) (Molenberghs and Verbeke, 2000) in which so-called random effects are added to correct for the dependence structure of the different measurements. A random effect enables the modeling of an individual development for each subject. LMMs can handle missing phenotype data under the assumption that the data are at least missing at random (MAR) (Molenberghs and Verbeke, 2000).

In the genetic context, these LMMs can be applied to perform longitudinal GWASs (Wendel et al., 2021). Using this, we previously (Wendel et al., 2021) investigated the genetic influence of individual SNPs on the course over time of executive functions, which control and coordinate mental processes. These GWASs demonstrated the versatility of LMMs in genetic association studies. Thus, the next step is to investigate pathways for association with longitudinal phenotypes, for example, the genomic basis of the longitudinal course of executive functions. For this, we can exploit that LMMs share an estimation equivalence with KMR models (Liu et al., 2007).

The aim of this work is to develop a longitudinal pathway analysis to test for the association between genetic factors and the longitudinal phenotype applying KMR and simultaneously allowing integrating network information. To be able to analyze longitudinal data, we extended KMR to long-KMR. Other authors have also studied longitudinal data (Yan et al., 2015; Ge et al., 2016; Wang et al., 2016) and created a KMR extension (Yan et al., 2015; Yan et al., 2018). However, in this extension only single genes can be tested for association (Yan et al., 2018) and these genes can solely be modeled with a weighted linear kernel. In our longitudinal pathway analysis, the whole pathway can be modeled with different kernels respectively prior to testing. For example, a linear kernel or a network-based kernel (Freytag et al., 2013), which enables the integration of network information in KMR can be applied. Moreover, different genetic effects including main, interaction, and joint genetic (main and interaction) effects can be considered. Thus, in long-KMR, we can model and test not only a main genetic effect, but most importantly also a genetic time-interaction effect. The latter translates to an association of the pathway with the trajectory of the considered phenotype.

In a simulation study, we assessed the properties of long-KMR regarding several aspects. We considered longitudinal studies with two and four measurement points. We compared the performance of long-KMR when applying a linear kernel or a network kernel. We also studied the influence of the pathway topology on the performance of the network kernel with a focus on the density of the pathway.

Finally, as a real-world application, we use long-KMR on the data from our previous longitudinal GWASs (Wendel et al., 2021) on executive functions of the PsyCourse Study (Budde et al., 2018). For this phenotype we chose several candidate pathways to be investigated with long-KMR.

In summary, in this paper we first present the theoretical aspects of long-KMR and the network kernel. We then describe the simulation approach used to evaluate our method, and, lastly, provide a real-world example of long-KMR.

## 2 Material and methods

In this section, we introduce the KMR analysis and its extension to analyze longitudinal data. We describe our simulation approach to investigate the type I error rate and power. Lastly, we present an application of long-KMR as example and give details on the PsyCourse Study data and the pathways used.

### 2.1 Kernel machine regression models

Let us assume $y_{i}$ is a quantitative phenotype for individual $i\;\left(i=1,\ldots ,\;n\right)$ with one measurement point per individual. We assume for the entire article that the pathway tested is represented as genotypes of the SNPs part of the pathway. The SNPs are coded as 0, 1, or 2, representing the number of minor alleles of the SNP in individual i . The genetic information for individual i of all selected SNPs s is stored as genotype vector $g_{i}$ . We regress $g_{i}$ on our phenotype of interest by applying the following model:

$$y_{i}=x_{i}\beta +h\left(g_{i}\right)+\varepsilon_{i},$$

where $y_{i}$ is the phenotype of interest for individual i , $x_{i}$ represents potential covariates, β is the regression coefficient vector, and h is a non-parametric function. This function $h\in H_{K}$ , where $H_{K}$ is a reproducing kernel Hilbert space with an inner product (Schaid, 2010; Ge et al., 2016). The reproducing kernel Hilbert space is generated by a positive semidefinite kernel function k (Liu et al., 2007; Ge et al., 2016). The mathematical characteristics of the reproducing kernel Hilbert space (e.g., inner product) allows approximating h as a linear combination of the kernel function k (Liu et al., 2007; Schaid, 2010; Ge et al., 2016). The “kernel trick” (Ge et al., 2016) specifies hereby that any positive semidefinite kernel function can be used as k . We define the corresponding kernel matrix K as $K≔k\left(g_{i},g_{j}\right)$ for any pair of individuals i and j of the associated kernel function k (Schaid, 2010; Ge et al., 2016). Here, we transform the high-dimensional n×s genotype matrix into a n×n similarity matrix. The kernel matrix K describes the similarity between each pair of individuals. By choosing a kernel, we can specify how to model the concept of genetic similarity. For example, we can use the popular linear kernel (LIN), which computes the similarity for each pair of individuals i and j by multiplying their genotype vectors $g_{i}$ and $g_{j}$ . The kernel matrix contains the elements $K\left(g_{i},g_{j}\right)=g_{i}^{T}g_{j}$ (matrix notation: $K=GG^{T}$ ). The linear kernel assumes that each SNP contributes a random independent value in an additive manner (Freytag et al., 2013). For the above model, we assume that the random error $\varepsilon_{i}∼N\left(0,\;\sigma_{\varepsilon }^{2}\right)$ and $h∼N\left(0,\;\tau K\right)$ with K being the kernel matrix and τ a variance component. The null hypothesis of our association test is $H_{0}:h=0$ being equivalent to $H_{0}:\;\tau =0$ (Liu et al., 2007; Wu et al., 2011). To test for association, we perform a variance component test (Liu et al., 2007; Wu et al., 2011).

The KMR model can be read as a LMM with h being interpreted as a random effect (Liu et al., 2007; Ge et al., 2016). The above model for a quantitative phenotype with one measurement per individual can also be described as LMM (in matrix notation) (Liu et al., 2007; Ge et al., 2016):

y=Xβ+h+ε,

where y is the vector of phenotypes for n individuals, X is the design matrix, β is the regression coefficient vector of the fixed effects, h $∼N\left(0,\;\tau K\right)$ is the random effect vector with K being the kernel matrix, the random error ε is normally distributed. The variance component test for this model (Liu et al., 2007; Ge et al., 2016) is:

$$Q_{cross}=\frac{1}{2\sigma_{\varepsilon }^{2}}\;{\left(y-X{\hat{\beta }}_{0}\right)}^{T}K\left(y-X{\hat{\beta }}_{0}\right),$$

where ${\hat{\beta }}_{0}$ are the estimates of the fixed effects under $H_{0}$ . For the longitudinal extension, we adjust for the dependence structure of the multiple measurements in the longitudinal data by including additional random effects (Molenberghs and Verbeke, 2000). Now we assume that $y_{i}$ is a quantitative longitudinal phenotype for individual $i\;\left(i=1,\ldots ,\;n\right)$ with m measurement points. The long-KMR model for individual i is:

$$y_{i}=X_{i}\beta +h\left(g_{i}\right)+Z_{i}b_{i}+\varepsilon_{i},$$

where $y_{i}$ is the phenotype vector of individual i , β is the fixed effect vector, and $b_{i}$ the random effect vector. We assume that only two random effects are added (random intercept and slope for time). Thus, we assume that $b_{i}∼N\left(0,D_{i}\right)$ with $D_{i}$ being a 2×2 covariance matrix and $\varepsilon_{i}∼N\left(0,\;R_{i}\right)$ with $R_{i}$ being a m×m covariance matrix, $b_{i}$ and $\varepsilon_{i}$ are uncorrelated. $X_{i}$ and $Z_{i}$ are two designs matrices for the fixed and random effects, respectively. The genotype vector $g_{i}$ and function h are given as above. To obtain the test statistic of the extended variance component test, we followed the steps proposed by (Liu et al., 2007; Yan et al., 2015). Therefore, we look at the longitudinal model in matrix notation considering the whole dataset:

$$y=X\beta +h\left(G\right)+Zb+\varepsilon$$

where y is the phenotype vector, $h∼N\left(0,\tau K\right)$ , $b∼N\left(0,D\right)$ ( $D=diag\left(D_{1},\ldots ,D_{n}\right)$ ) and $\varepsilon ∼N\left(0,\;R\right)$ with $R=diag\left(R_{1},\ldots ,R_{n}\right)$ . The design matrices are $X={\left(X_{1},\ldots ,X_{n}\right)}^{T}$ for the fixed effects and $Z=diag\left(Z_{1},\ldots ,Z_{n}\right)$ for the random effects, with β and b being the fixed and random effect vectors, respectively. The null hypothesis remains $H_{0}:\;\tau =0$ . The altered test statistic is:

$$Q_{long}=\frac{1}{2}{\left(y-X{\hat{\beta }}_{0}\right)}^{T}{\hat{\Sigma }}_{0}^{-1}K{\hat{\Sigma }}_{0}^{-1}\left(y-X{\hat{\beta }}_{0}\right),$$

where ${\hat{\beta }}_{0}$ are the estimates of the fixed effects under $H_{0}$ and ${\hat{\Sigma }}_{0}^{-1}$ are the inverse of the covariance-variance matrix under $H_{0}$ with ${\hat{\Sigma }}_{0}={\hat{R}}_{0}+Z{\hat{D}}_{0}Z^{T}$ . The test statistic is a quadratic form and follows a mixture of $\chi^{2}$ distributions with $Q_{long}∼\overset{L}{\underset{l=1}{\sum }}\lambda_{l}\chi_{1}^{2}$ , where $\lambda_{l}$ are the eigenvalues of $\frac{1}{2}\;V_{0}^{\frac{1}{2}}{\hat{\Sigma }}_{0}^{-1}K{\hat{\Sigma }}_{0}^{-1}V_{0}^{\frac{1}{2}}$ with $V_{0}={\hat{\Sigma }}_{0}-X{\left(X^{T}{\hat{\Sigma }}_{0}^{-1}X\right)}^{-1}X^{T}$ (Yan et al., 2015; Ge et al., 2016). We computed the *p*-values with the Davies method (Davies, 1980).

Next, we will apply long-KMR to test a genetic ( G ) interaction effect with time ( t ). Here, we multiply the time vector of individual $i,\;t_{i}=0,\ldots ,\;m-1$ with the genotype vector $g_{i}$ of individual i . In addition to the main genetic kernel ( $h\left(G\right)$ ) this extended model contains a kernel modelling the genetic time interaction effect ( t×G , further denoted as time-interaction effect). In matrix notation (whole dataset) the model is:

$$y=X\beta +h_{1}\left(G\right)+h_{2}\left(t\times G\right)+Zb+\varepsilon ,$$

where $\varepsilon ∼N\left(0,R\right)$ , $h_{1}\left(G\right)∼N\left(0,\tau_{1}K_{1}\right)$ and $h_{2}\left(t\times G\right)∼N\left(0,\tau_{2}K_{2}\right)$ . The notation follows the previous long-KMR in matrix notation. When fitting the LMM in this interaction model, we have to integrate $K_{1}$ as random effect in form of a variance-covariance matrix. This is complex and computationally very extensive. We use two different approaches to reduce the computation time. For the first approach, we only include $h_{2}\left(t\times G\right)$ in our model without adjusting for the main genetic effect ( $h_{1}\left(G\right)$ ) altering the LMM independent of any kernel matrix under the null hypothesis. For the second approach, we adjust for the main genetic effect by performing a principal component analysis (PCA) on $K_{1}$ . This so-called kernel principal component analysis (KPCA, (Schölkopf et al., 1997; Schölkopf et al., 1998)) has been previously applied in different situations (Schölkopf et al., 1997; Schölkopf et al., 1998; Gao et al., 2011). We replace $h_{1}\left(G\right)$ by a number of top principal components, which are added as fixed effects. By only including additional fixed effects, we avoid complex variance structures while adjusting for the main genetic effect. In both approaches, we are interested in testing $K_{2}$ **,** modeling the time-interaction effect for association. The null hypothesis is defined as $H_{0}:\tau_{2}=0$ . The test statistic of long-KMR is slightly altered, as K of $Q_{long}$ is exchanged with $K_{2}$ modeling the time-interaction effect.

### 2.2 Network kernel

In long-KMR, we can also integrate network information on the studied pathway by applying the network-based kernel (Freytag et al., 2013) (noted as network kernel in the following). The network kernel is defined as $K=GANA^{T}G^{T}$ , where *G* is the genotype matrix with the genotypes for each individual, A is an annotation matrix and *N* is an adjacency matrix of the pathway. The annotation matrix contains elements $a_{\rho \gamma }\in \left(0,1\right)$ describing whether a SNP ρ ( ρ = 1, …, s ) is mapped to the gene γ (=1) or not (=0). The assignment of a SNP to a gene is defined by its genomic location. We can adjust for different gene sizes (= number of SNPs mapped) by dividing $a_{\rho \gamma }$ by the square root of the number of SNPs mapped to gene $\gamma \;\left(Freytag\;et\;al.,\;2013\right)$ . The size-adjusted annotation matrix replaces A in the network kernel. We distinguish these network kernels by denoting the unadjusted kernel as NET and the size-adjusted network kernel as ANET [similar to (Freytag et al., 2013)]. The elements of the quadratic adjacency matrix for a pathway are ${\;n}_{\gamma \gamma^{\prime }}=1$ , if genes γ and $\gamma^{\prime }$ interact with each other, or zero otherwise. By definition (Freytag et al., 2013), the genes all interact with themselves; thus, the main diagonal of N contains only ‘1’s. We do not distinguish between the different types of gene interaction (e.g., activation and inhibition) owing to the characteristics of the studied pathways (more details later). We slightly modify the network (topology) of the pathway to ensure a positive semidefinite kernel. We do not describe the details of these modifications here; please refer to (Freytag et al., 2013) for more details.

### 2.3 Simulation study

We studied type I error rates and power in different scenarios to assess the performance of long-KMR for different genetic effects and the network kernel. The type I error rate is defined as the proportion of simulations that have a *p*-value < α in the simulations of the model with no genetic effects (null model). Here we set α to equal 5%, 1%, 0.5%, and 0.1%, respectively. In the scenarios in which we simulated genetic effects, we determined the power as the proportion of simulations with a *p*-value <5% threshold. In total, we compared the power for three different genetic effect models. We simulated two single-effect models containing either a main genetic effect or a time-interaction effect. We also created a more complex model, the joint model, which comprises a main genetic effect and a time-interaction effect. The joint model was only studied in a limited number of scenarios, as portrayed in Table 1. For the single-effect models, we had the same scenarios to evaluate the type I error rates and the power. We assessed the influence of the number of measurement points comparing two-measurement models with four-measurement models. The type I error rates and respective power of the linear kernel (LIN) and the network kernel (NET) were compared. For the latter, we only used the unadjusted network kernel (NET), as all genes had the same size. For two measurement points representing a pre/post-analysis, we applied the ANCOVA model (Table 1) to compare their performances with long-KMR. For the four-measurement models, we compared the performance of long-KMR with the previously published KMgene package (Yan et al., 2018). Further, we compared the analysis of complete phenotype data with incomplete phenotype data with 25% or 50% of the data missing [assuming missing at random mechanism (MAR)].

**TABLE 1:** Models of simulation study.

Model names	Kernel	Statistical model (without genetic effect)	Phenotype data	Genetic effect model		
				Main genetic	Time- interaction	Joint
Two-measurement models						
KMR-LIN-ANCOVA	Linear kernel	ANCOVA: $y_{2}=\beta_{0}X_{i}+\beta_{1}y_{1}+\varepsilon$	complete data	*	*	—
KMR-LIN-m2	Linear kernel	KMR (LMM): y=Xβ+Zb+ε	complete data	*	*	—
KMR-LIN-m2_25MAR			25% missing data (MAR)	*	*	—
KMR-NET-d0.8-m2	Network kernel (pathway d = 0.8)	KMR (LMM): y=Xβ+Zb+ε	complete data	*	*	—
KMR-NET-d0.8-m2_25MAR			25% missing data (MAR)	*	*	—
KMR-NET-d0.5-m2	Network kernel (pathway d = 0.5)	KMR (LMM): y=Xβ+Zb+ε	complete data	*	*	—
KMR-NET-d0.5-m2_25MAR			25% missing data (MAR)	*	*	—
KMR-NET-d0.2-m2	Network kernel (pathway d = 0.2)	KMR (LMM): y=Xβ+Zb+ε	complete data	*	*	—
KMR-NET-d0.2-m2_25MAR			25% missing data (MAR)	*	*	—
Four-measurement models						
KMR-LIN-m4	Linear kernel	KMR (LMM): y=Xβ+Zb+ε	complete data	*	*	*
KMR-LIN-m4_25MAR			25% missing data (MAR)	*	*	—
KMR-LIN-m4_50MAR			50% missing data (MAR)	*	*	—
KMR-NET-d0.8-m4	Network kernel (pathway d = 0.8)	KMR (LMM): y=Xβ+Zb+ε	complete data	*	*	*
KMR-NET-d0.8-m4_25MAR			25% missing data (MAR)	*	*	—
KMR-NET-d0.8-m4_50MAR			50% missing data (MAR)	*	*	—
KMR-NET-d0.5-m4	Network kernel (pathway d = 0.5)	KMR (LMM): y=Xβ+Zb+ε	complete data	*	*	*
KMR-NET-d0.5-m4_25MAR			25% missing data (MAR)	*	*	—
KMR-NET-d0.5-m4_50MAR			50% missing data (MAR)	*	*	—
KMR-NET-d0.2-m4	Network kernel (pathway d = 0.2)	KMR (LMM): y=Xβ+Zb+ε	complete data	*	*	*
KMR-NET-d0.2-m4_25MAR			25% missing data (MAR)	*	*	—
KMR-NET-d0.2-m4_50MAR			50% missing data (MAR)	*	*	—
KMgene (comparison model) (Yan et al., 2018)	Weighted linear kernel	KMR (LMM): y=Xβ+Zb+ε	complete data	*	—	—

For each model, the kernel, the applied statistical models and the used phenotype data sets are displayed. For network kernel the pathway density (d) is given. The phenotype data can be complete or with 25/50% of values missing at random (MAR). The addressed genetic effects (main genetic, time-interaction and joint effect) are indicated with an asterisk “*”.

**FIGURE 1:**
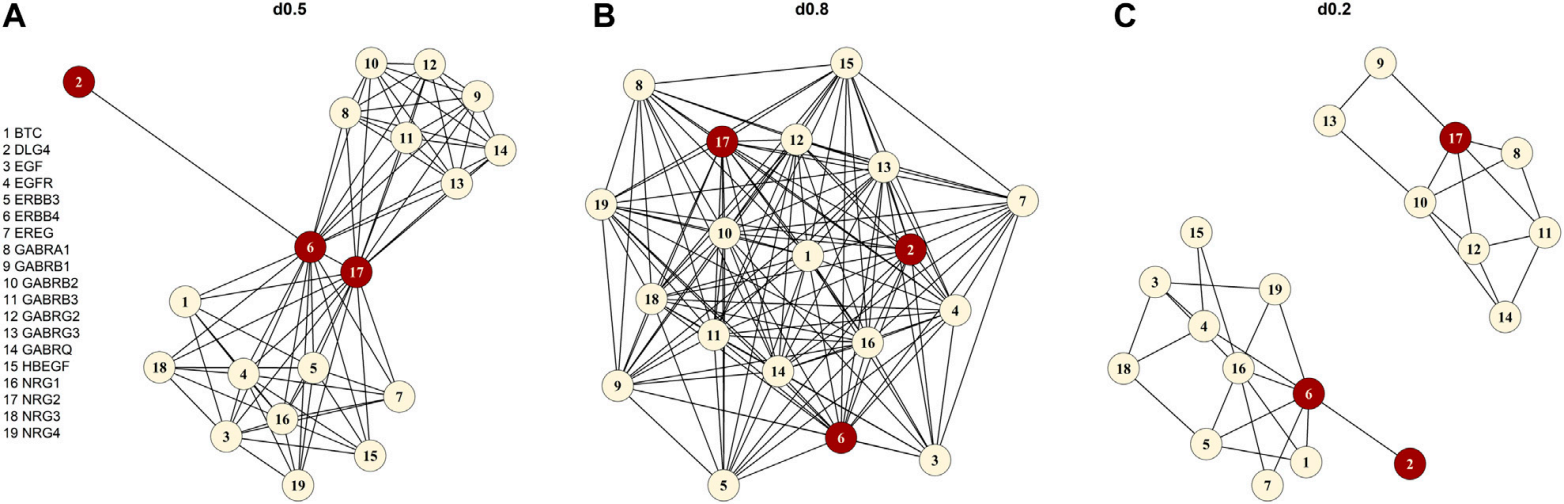
Graphical illustration of the “signaling by ERBB4” pathway **(A)** original form without the chemical compound (d0.5), **(B)** the simulated high-density pathway (d0.8), **(C)** the artificially created low-density pathway (d0.2). The red vertices are the three defined causal genes (NRG2, ERBB4 and DLG4).

To evaluate the performance of the network kernel on pathways with different characteristics, we focused on the density (=d) of a pathway. This density is a graph-theoretical characteristic defined as the ratio of the number of present connections divided by the maximum number of possible connections in a pathway (d $\in \left[0,1\right]$ ). When we consider a pathway as a graph in which the genes are the nodes and the connections of the genes are the edges linking the nodes, the density can be computed straightforwardly. We determined the density of the original pathway after downloading the pathway from the Reactome database, applying the *igraph* package (Csardi and Nepusz, 2006). We selected the “signaling by ERBB4” pathway [R-HSA-1236394, (Stern, 2019)] as foundation pathway for our simulation study. The selection process for “signaling by ERBB4” is described in detail in the section *Pathway Data*. The “signaling by ERBB4” pathway has a density of 0.46 but we denoted the pathway as d0.5 after rounding up *d* = 0.46 (Figure 1A). In addition, we created two artificial pathway topologies with different density originating from the original “signaling by ERBB4” pathway. We generated a high-density pathway with *d* = 0.81 (denoted as d0.8, Figure 1B) and a low-density pathway with *d* = 0.20 (d0.2, Figure 1C). Table 1 lists all the models studied with an overview of the different settings.

We sampled genotypes for 10,000 individuals with HAPGEN2 (Su et al., 2011) using common (MAF ≥ 0.05) variants of chromosome one of the CEU sample of the International HapMap Project (HapMap 3 release 2) (Altshuler et al. 2010). In analogy to our foundation pathway “signaling by ERBB4” with 19 genes (Figure 1), we created 19 “pseudo-” genes all with a size of 50 SNPs (in total: 950 SNPs). The 950 SNPs were simulated in the region between 742 kbp and 112,709 kbp with a separation of 500 kbp between SNPs of the single “pseudo-” genes to prevent LD. We assign the simulated SNPs to a “pseudo-” gene. For each simulation setting, we created 100 smaller genotype matrices each containing 950 SNPs and 1,000 individuals. To achieve this, we randomly drew genotypes for 1,000 individuals from the previously simulated 10,000 individual sample (elementary matrix). For each of the 100 genotype matrices, we simulated 1,000 quantitative phenotypes according to the LMM below, resulting in a total number of 100,000 replications [similar to (Yan et al., 2015)].

For the null model corresponding to the null hypothesis of no genetic effects, we simulated the quantitative phenotypes according to the following LMM for an individual i ( i=1, …, 1000 ):

$$y_{i}=0.5*\;X_{1i}+0.25*X_{2i}+0.2*t_{i}+u_{i},$$

where $X_{1i}$ is a binary time-invariant variable with a probability of 0.5 (e.g., sex of individual i ), $X_{2i}$ is normally distributed and time-invariant with $N\left(50,5\right)$ (e.g., age at first measurement point) and $t_{i}=0,\;\ldots ,\;m-1$ where m equals the total number of measurement points ( m = 2 or m = 4). Random error and random effects are modelled by $u_{i}$ , which follow a multivariate normal distribution with mean zero and $Var\left(y_{i}\right)$ . $Var\left(y_{i}\right)$ is defined as follows:

$$Var\left(y_{i}\right)=Z_{i}\;\left(\begin{array}{cc} \sigma_{intercept}^{2} & \sigma_{cov}\\ \sigma_{cov} & \sigma_{time}^{2} \end{array}\right)Z_{i}^{T}+\sigma_{\varepsilon }^{2}I_{m\times m}$$

where $I_{m\times m}$ is the identity matrix, $\sigma_{intercept}^{2}=\sigma_{time}^{2}=\sigma_{\varepsilon }^{2}=1$ and $\sigma_{cov}=-0.5$ . We selected the parameters similarly to (Yan et al., 2015). For the missing phenotype simulations, we assumed MAR and generated the missing phenotypes with the R package mice (van Buuren and Groothuis-Oudshoorn, 2011 *)*.

For the power simulations, we added genetic effects to our null model to simulate the phenotypes. All models comprised three causal “pseudo-” genes each with three causal SNPs (in total: nine causal SNPs). The effect sizes $\beta_{k}$ for each SNP had the same value. The effect size for the joint model was 0.04. For the single-effect models, we studied three different scenarios with three different effect sizes β=0.04, 0.06, and 0.08 . To compare the different network topologies, we defined the genes NRG2, ERBB4, and DLG4 of the “signaling by ERBB4” pathway as the causal genes (red nodes, Figure 1) based on their central position in the pathway. The main genetic effect model adds a sum consisting of the additive effect of the causal SNPs to the phenotype ( $\overset{9}{\underset{k=1}{\sum }}\beta_{k}{*SNP}_{ik}$ ) for each individual i . The time-interaction effect includes only the sum of the product of the causal SNPs and the time ( $\overset{9}{\underset{k=1}{\sum }}\beta_{k}*\left({SNP}_{ik}*t_{ij}\right)$ ) at each time point j for individual i . The joint model comprised both sums ( $\overset{9}{\underset{k=1}{\sum }}\beta_{k}*{SNP}_{ik}+\overset{9}{\underset{k=1}{\sum }}\beta_{k}*\left({SNP}_{ik}*t_{ij}\right)$ ). In the first model, the main genetic kernel ( $h\left(G\right)$ ) is tested. The latter models test the time-interaction kernel ( $h\left(t\times G\right)$ ) for association. In the joint model, the main genetic kernel was computed with the linear kernel. Here, we performed a principal component analysis on the main genetic kernel to adjust for the main genetic effect to simplify computational complexity and gain speed. We added the top two principal components as fixed effects to our model.

To compare the type I error rate and power of long-KMR with KMgene (Yan et al., 2015; Yan et al., 2018) we performed a simulation with KMgene for 1,000 individuals and four measurement points. Here, only the main genetic effect model was simulated because of the characteristics of KMgene (Yan et al., 2018). For every simulated gene (in total: 19, each with 50 SNPs), we obtained a gene-level *p*-value, which we combined with the Fisher’s method (Fisher, 1925; Larson et al., 2017) to receive a pathway *p*-value. This *p*-value combination was performed with the R package *metap* (Dewey, 2022).

### 2.4 Application to real data

#### 2.4.1 The PsyCourse Study

The PsyCourse Study is a longitudinal, multi-center study comprising patients with diagnoses from the affective-to-psychotic spectrum and neurotypical individuals. A large battery of different phenotypes, including demographics, cognition, self- and observer rating scales, are assessed at up to four measurement points each 6 months apart (Budde et al., 2018). For our application, we analyzed 1,594 genotyped individuals including patients from the affective-to-psychotic spectrum (411 bipolar I disorder, 113 bipolar II disorder, 466 schizophrenia, 90 schizoaffective disorder, 10 schizophreniform disorder, 6 brief psychotic disorder and 94 with recurrent depression) and 404 control individuals. The diagnoses were determined according to the criteria in the Diagnostic and Statistical Manual of Mental Disorders, Fourth Edition (DSM-IV); a subset of individuals suffering from schizophrenia (45 individuals) was diagnosed according to ICD-10 criteria. Different centers in Germany and Austria conducted the recruitment of the study participants. All individuals provided written informed consent, and the study protocol was approved by the respective ethics committees at each study center [see ref. (Budde et al., 2018)]. Based on their symptoms, the individuals were broadly distinguished into an “affective” group (618, predominantly affective symptoms including bipolar disorder I and II and recurrent depression) or a “psychotic” group (572, predominantly psychotic symptoms encompassing schizophrenia, schizoaffective, schizophreniform, and brief psychotic disorder).

As phenotype of interest, we chose the Trail Making Test, part B (TMT-B) (Bowie and Harvey, 2006). TMT-B is applied to assess set-shifting, one of the three latent core skills of executive functions (Diamond, 2013; Friedman et al., 2016), a specific group of cognitive abilities. During the test, an individual is required to connect numbers (numbers: 1–26) and letters of the alphabet in ascending alternating order, for which the time (in seconds) to finish this task is measured to represent the test score. Study participants with a time >300 s were set to 300 s according to the recommendation by (Strauss et al., 2006). The higher the TMT-B score of an individual is, the greater the cognitive impairment.

Genotyping was performed with the Illumina Infinium Global Screening Array-24 Kit (version 3.0 or version 1.0) and the imputation took place on the Michigan imputation server (Das et al., 2016) with the haplotype reference consortium as reference panel. Quality control (QC) steps were performed according to standard procedures described elsewhere (Smigielski et al., 2021). In the analysis, we included approximately 3.5 million imputed SNPs with a MAF >0.05. We used PLINK v1.9 (Chang et al., 2015) (https://www.cog-genomics.org/plink/) to compute the ancestry principal components.

#### 2.4.2 Pathway data

We focused on pathways on the Reactome database (Jassal et al., 2019) downloaded from Pathway Commons database Version 12 (Rodchenkov et al., 2019) (Reactome version 69, date: 01|14|22). First, we selected pathways based on different keywords connected to executive functions including dopamine, serotonin, GABA, glutamate, NDMA, synaptic, voltage-gated potassium channels, plasticity, and prefrontal cortex. The keywords resulted in 130 pathways, which we reduced to the 17 pathways finally studied (Table 2). We selected the 17 pathways according to different criteria. First, we only used pathways that we were able to download. The pathway had to be between 15 and 100 genes in size, and the number of chemical compounds (CHEBI) in the pathway had to be at most five. For each pathway, we specified the density (d) by applying the *igraph* package (Csardi and Nepusz, 2006) and included only pathways with d ≤ 0.95. The 17 selected pathways with specific characteristics e.g., number of genes, density, and average degree are displayed in Table 2. The average degree of a pathway is the average number of connections of a gene (=node). Originating from the list of 17 pathways, we chose “signaling by ERBB4” (Stern, 2019) (https://reactome.org/content/detail/R-HSA-1236394) as foundation pathway for our simulation study. This pathway was selected for its moderate size of 19 genes and because it only contains one CHEBI. The network consists of only one graph component, also denoted as connected (i.e., any gene can be reached from any other gene *via* a path). Most importantly, the pathway has an intermediate density of 0.46, which was a good basis for further artificial pathways we generated with high and low densities. “Signaling by ERBB4” is connected to schizophrenia (Banerjee et al., 2010) and schizophrenia endophenotypes, e.g. cognitive functions (Banerjee et al., 2010; Tian et al., 2017; Shi and Bergson, 2020) and thus is biologically very interesting. We deleted the CHEBI, as SNPs are the genomic basis in our analysis and a CHEBI cannot be assigned.

**TABLE 2:** Selected pathways investigated in the real-data example.

Pathway name	Reactome identifier (R-HSA-xxx)	URL	Pathway Characteristics		
			No. Genes	Average degree	Density (d)
NCAM1 interactions	419037	https://reactome.org/content/detail/R-HSA-419037	37	3.40	0.093
Receptor-type tyrosine-protein phosphatases	388844	https://reactome.org/content/detail/R-HSA-388844	20	3.10	0.163
MECP2 regulates neuronal receptors and channels	9022699	https://reactome.org/content/detail/R-HSA-9022699	18	3.00	0.177
EPHB-mediated forward signaling	3928662	https://reactome.org/content/detail/R-HSA-3928662	33	7.45	0.233
Synaptic adhesion-like molecules*	8849932	https://reactome.org/content/detail/R-HSA-8849932	22	4.67	0.233
Transcriptional Regulation by MECP2	8986944	https://reactome.org/content/detail/R-HSA-8986944	17	4.00	0.250
Neurexins and neuroligins	6794361	https://reactome.org/content/detail/R-HSA-6794361	57	14.39	0.257
EPH-Ephrin signaling	2682334	https://reactome.org/content/detail/R-HSA-2682334	22	5.45	0.260
Regulation of MECP2 expression and activity	9022692	https://reactome.org/content/detail/R-HSA-9022692	31	8.00	0.267
Signaling by ERBB4*	1236394	https://reactome.org/content/detail/R-HSA-1236394	19	8.32	0.462
Trafficking of AMPA receptors	399719	https://reactome.org/content/detail/R-HSA-399719	17	7.88	0.493
NCAM signaling for neurite out-growth*	375165	https://reactome.org/content/detail/R-HSA-375165	21	11.00	0.524
Assembly and cell surface presentation of NMDA receptors	9609736	https://reactome.org/content/detail/R-HSA-9609736	24	12.08	0.525
Interaction between L1 and Ankyrins	445095	https://reactome.org/content/detail/R-HSA-445095	29	20.28	0.724
Negative regulation of NMDA receptor-mediated neuronal transmission	9617324	https://reactome.org/content/detail/R-HSA-9617324	21	16.86	0.843
Long-term potentiation*	9620244	https://reactome.org/content/detail/R-HSA-9620244	23	19.22	0.874
Ion channel transport*	983712	https://reactome.org/content/detail/R-HSA-983712	24	21.83	0.949

The pathways are listed according to ascending density and with links to their Reactome entry. The foundation pathway in our simulation study is printed in bold, the pathways with a *p*-value <0.1 in our application are further discussed and are labelled with an asterisk “*“.

#### 2.4.3 Statistical analysis

Each of the 17 pathways was tested for association with TMT-B. To fulfil the normality assumption, the TMT-B was log-transformed (lgTMT-B). We included the following fixed effects in the model: sex, age at first measurement point, diagnostic group (affective, psychotic, and control), time, and the top five ancestry principal components. A random intercept and a random slope for the time effect were also added. We tested each pathway for a potential main genetic and a time-interaction effect. The linear kernel (LIN), the unadjusted network (NET), and the size-adjusted network kernel (ANET) were applied. We assigned a SNP to a gene of a pathway based on its genomic location with a mapping window of ± 500 kbp on each side of the gene. For the multiple testing correction, we considered the overlap of the tested pathways and computed the number of effective pathways ( $P_{eff}$ ) according to (Hendricks et al., 2013; Larson et al., 2017). We computed a 17 × 325 matrix W for the 17 tested pathways and the 325 genes comprised in the 17 pathways with

$$w_{r\gamma }=\left\{\begin{array}{c} \frac{1}{\sqrt{\left|P_{r}\right|}},\;if\;gene\;\gamma \in pathway\;r\\ 0,\;otherwise \end{array}\right.,$$

where $\left|P_{r}\right|$ is the number of genes contained in pathway r . From the product of this matrix with its transpose, we computed the eigenvalues to obtain $P_{eff}$ according to the Gao approach (Hendricks et al., 2013). We determined the number of eigenvalues required to fulfil $\frac{\overset{P_{eff}}{\underset{r=1}{\sum }}\left|\lambda_{r}\right|}{\overset{P}{\underset{r=1}{\sum }}\left|\lambda_{r}\right|}\geq c$ , setting c to 0.95 leading to $P_{eff}=15$ . We set c to 0.95, as it was sufficient for us that the effective number of pathways explains 95% of the total variance. The adjusted significance level was computed as $\alpha_{Gao}=\frac{0.05}{15}=0.0033$ .

### 2.5 Code availability

We performed all analyses with R (R Core Team, 2021), which we also used to implement the KMR for quantitative longitudinal data and cross-sectional binary and quantitative data as an R package kalpra (kernel approach for longitudinal pathway regression analysis) available at https://gitlab.gwdg.de/bernadette.wendel/kalpra. In addition to the linear and network kernels, a quadratic kernel is also available. The pathway information can be directly downloaded and transformed into an annotation and adjacency matrix. The computational aspects for some example analyses are provided in Supplementary Table S1.

## 3 Results

### 3.1 Simulation studies

**FIGURE 2:**
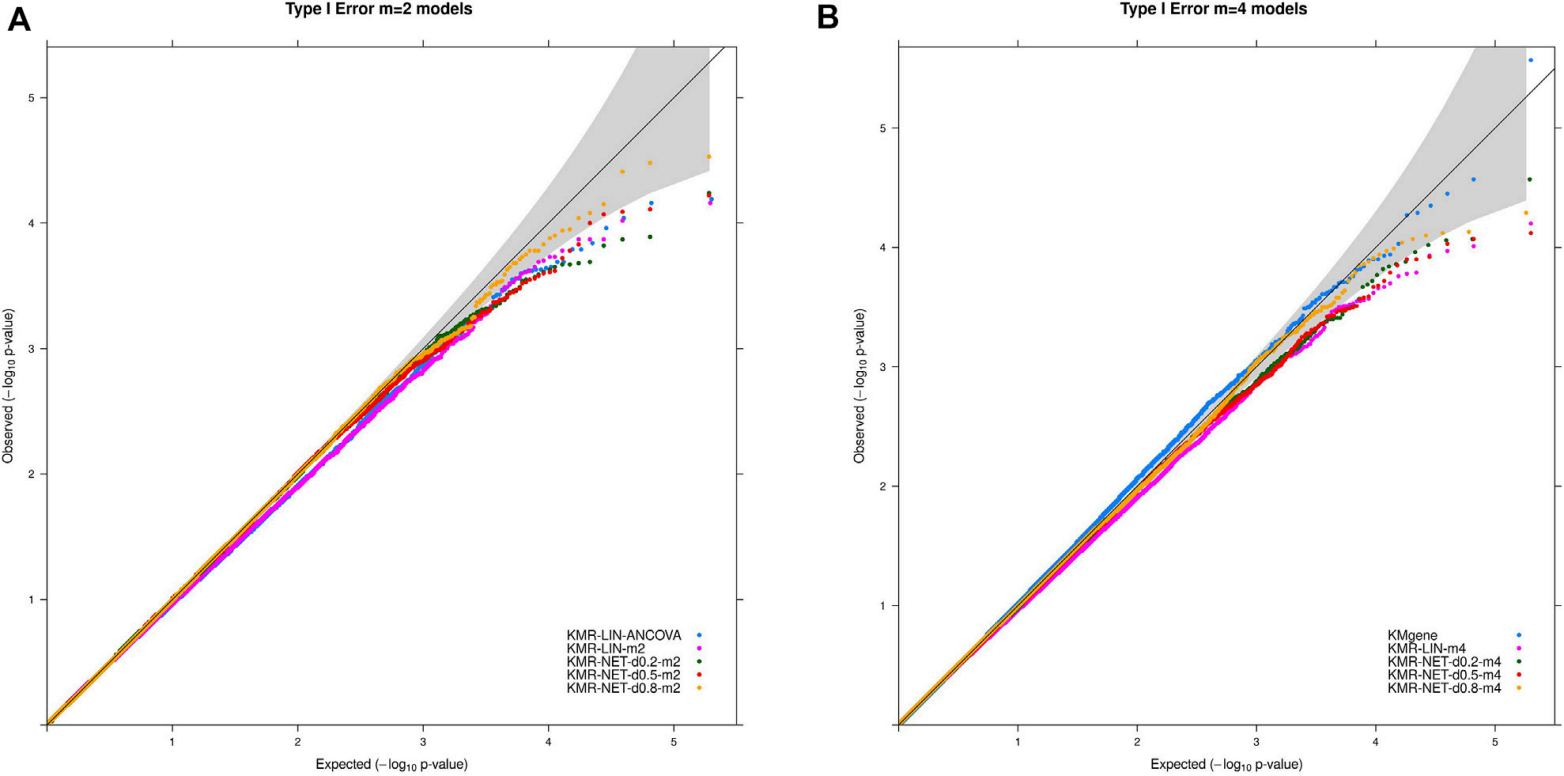
QQ-plots for the type I error rate for our simulation studies divided for the **(A)** two-measurement and **(B)** four-measurement models and the comparison model KMgene.

The type I error rate in our simulation study is defined, as mentioned above, as the proportion of simulations for which we obtained a *p*-value < α (*α* = 5%, 1%, 0.5%, and 0.1%) in the null simulations without genetic effects. The type I error rates were maintained overall at the different α thresholds for the models in our simulation scenarios. We did detect individual type I error rates only slightly exceeding the respective significance levels, e.g., 5% and 1%, for three models (KMR-NET-d0.5-m2, KMR-NET-d0.8-m2, and KMR-NET-d0.8-m4). However, all the values lie in the range of expected random variations (confidence intervals of the null model simulations carried out, data not shown). KMR-NET-d0.8-m2 presented the largest increase in type I error of 5.09% at the significance level of 5%. Table 3 displays the type I error rates. The error rates of the different network kernels (all densities) were overall higher compared to the linear kernel and were closer to the nominal level. The combined pathway *p*-values of the KMgene analysis revealed an inflation of the error rates. The error rates for the analyses of the missing aspect for the different network kernel were also maintained (Supplementary Table S2). Figure 2 displays a QQ-plot of the distribution of the multiple error rates for all models analyzing complete data distinguished between the two-measurement and four-measurement models including KMgene.

**TABLE 3:** Type I error rates of the simulation studies.

Models	Estimated type I error rate (%)			
	*α* = 5%	*α* = 1%	*α* = 0.5%	*α* = 0.1%
KMR-LIN-ANCOVA	4.33	0.79	0.37	0.07
KMR-LIN-m2	4.37	0.78	0.38	0.07
KMR-NET-d0.8-m2	5.09	1.00	0.50	0.09
KMR-NET-d0.5-m2	5.02	1.01	0.48	0.07
KMR-NET-d0.2-m2	4.96	0.96	0.50	0.09
KMR-LIN-m4_25MAR	4.47	0.84	0.40	0.08
KMR-LIN-m4_50MAR	4.38	0.82	0.41	0.09
KMR-LIN-m4	4.32	0.78	0.38	0.07
KMR-NET-d0.8-m4	4.93	0.92	0.44	0.11
KMR-NET-d0.5-m4	4.83	0.92	0.45	0.07
KMR-NET-d0.2-m4	4.48	0.94	0.45	0.08
KMgene* Yan et al. (2018)	5.31	1.13	0.57	0.11

Simulated type I error for tests at significance levels of *α* = 5%, 1%, 0.5% and 0.1% are displayed. The simulations are based on 100,000 runs each with 1,000 individuals. *For comparability, the single gene-level *p*-values of KMgene are combined to a pathway *p*-value using Fisher’s method.

**FIGURE 3:**
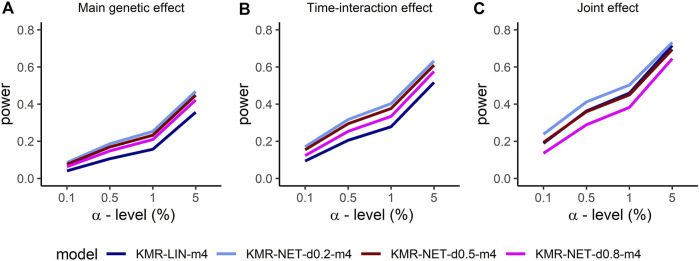
Comparison of the power for the four-measurement models (KMR-LIN, KMR-NET-d0.2, KMR-NET-d0.5, KMR-NET-d0.8) with complete phenotype data and effect size *β* = 0.04 for the different genetic effect models with **(A)** the main genetic **(B)** the time-interaction and **(C)** the joint effect in our simulation study.

For the two single-genetic-effect models (main genetic and time-interaction model), the power comparison of the two-measurement models revealed that the LMM had the highest power independent of effect size and for either kernel. ANCOVA had the lowest power for the two-measurement models in comparison. Table 4 displays the results for the effect size *β* = 0.04. Increasing the number of measurement points resulted in an improvement of the power for long-KMR, in particular for the time-interaction effect (an increase from 21% to 52% (time-interaction effect) compared to 33%–36% (main genetic effect)). Overall, the time-interaction effect yielded a higher power for the four-measurement models, especially for the smaller effect sizes 0.04 (Table 4) and 0.06 (Supplementary Table S3). An additional power benefit compared to the linear kernel was achieved when applying the network kernel. For the main genetic effect with effect size 0.04, the network kernel in the two-measurement models demonstrate a higher power than KMR-LIN-m4. However, the power gain for the network kernel depends on the pathway density. The power increases with decreasing density (d0.2 > d0.5 > d0.8). A direct comparison of the power for the linear and network kernels for the four-measurement models is displayed in Figures 3A,B for the main genetic effect and the time-interaction effect, respectively. The power differences between KMR-LIN-m4 and KMR-NET-d0.8-m4, the pathway with the highest density, fluctuated in the different settings (Table 4; Supplementary Tables S3,S4). In the joint modeling of main genetic and time-interaction effects, the network kernel with the lowest density displayed the highest power. Table 5 illustrates the results for a genetic effect size of 0.04. Here, at the significance level of 5% the linear kernel had the second highest power followed by KMR-NET-d0.5-m4 and KMR-NET-d0.8-m4 (Table 5). As displayed in Figure 3C, the power of LIN-m4 and KMR-NET-d0.5-m4 are very similar at different significance levels.

**TABLE 4:** Power results of the simulation study.

	Genetic effect					
	Main genetic effect			Time-interaction effect		
Models	Complete	25MAR	50MAR	Complete	25MAR	50MAR
KMR-LIN-ANCOVA	12.48% [12.28; 12.69]	—	—	5.16% [05.02; 05.30]	—	—
KMR-LIN-m2	33.09% [32.79; 33.38]	25.26% [24.99; 25.53]	—	21.28% [21.02; 21.53]	07.12% [06.96; 07.28]	—
KMR-NET-d0.8-m2	39.46% [39.15; 39.76]	31.66% [31.37; 31.95]	—	27.14% [26.85; 27.43]	18.92% [18.68; 19.17]	—
KMR-NET-d0.5-m2	42.10% [41.78; 42.41]	33.70% [33.41; 33.99]	—	28.68% [28.38; 28.97]	19.87% [19.62; 20.12]	—
KMR-NET-d0.2-m2	43.68% [43.36; 43.99]	35.18% [34.88; 35.48]	—	29.81% [29.49; 30.12]	20.68% [20.43; 20.94]	—
KMR-LIN-m4	35.68% [35.38; 35.96]	31.00% [30.72; 31.29]	23.10% [22.83; 23.36]	51.72% [51.41; 52.03]	44.61% [44.30; 44.91]	31.36% [31.07; 31.65]
KMR-NET-d0.8-m4	42.24% [41.93; 42.56]	37.66% [37.36; 37.96]	29.42% [29.14; 29.70]	57.62% [57.32; 57.93]	50.66% [50.35; 50.97]	38.57% [38.27; 38.87]
KMR-NET-d0.5-m4	44.83% [44.51; 45.15]	39.86% [39.56; 40.16]	31.21% [30.93; 31.50]	61.05% [60.75; 61.35]	54.10% [53.79; 54.41]	40.77% [40.47; 41.08]
KMR-NET-d0.2-m4	46.86% [46.54; 47.18]	41.76% [41.46; 42.06]	32.87% [32.58; 33.16]	63.28% [62.98; 63.59]	56.12% [55.81; 56.43]	42.43% [42.13; 42.74]
KMgene* (Yan et al., 2018)	31.02% [30.74; 31.31]	—	—	—	—	—

Simulated power to detect an effect of size 0.04 with a test at significance levels of *α* = 5% is displayed. The simulations are based on 100,000 runs each with 1,000 individuals. Power estimates together with 95% confidence interval are presented for genetic main and time-interaction effects. Phenotype data were either complete or with 25/50% of values missing at random (MAR). Model names correspond with Table 1.

*For comparability, the single gene-level *p*-values of KMgene are combined to a pathway *p*-value using Fisher’s method.

**TABLE 5:** Power comparison for main, time-interaction and joint effects.

Models	Genetic effect		
	Main genetic effect	Time-interaction effect	Joint effect
KMR-LIN-m4	35.68% [35.38; 35.96]	51.72% [51.41; 52.03]	71.55% [71.27; 71.83]
KMR-NET-d0.8-m4	42.24% [41.93; 42.56]	57.62% [57.32; 57.93]	64.47% [64.16; 64.78]
KMR-NET-d0.5-m4	44.83% [44.51; 45.15]	61.05% [60.75; 61.35]	69.44% [69.14; 69.74]
KMR-NET-d0.2-m4	46.86% [46.54; 47.18]	63.28% [62.98; 63.59]	73.23% [72.95; 73.50]

Simulated power to detect an effect of size 0.04 with a test at significance levels of *α* = 5% is displayed. The simulations are based on 100,000 runs each with 1,000 individuals. Power estimates together with 95% confidence interval are presented for genetic main, time-interaction and joint effects. Model names correspond with Table 1. The adjustment for the main genetic effect in the joint genetic effect was performed by adding the top two principal components of a PCA on a main linear kernel.

The analyses performed with different percentages of missing data revealed similar features for the single-effect models, with a general decrease of power compared to the analysis of a complete phenotype data set (Table 4). In general, the power increased with increasing effect sizes (*β* = 0.04, 0.06, and 0.08). For example, for KMR-LIN-m4 testing the main genetic effect, the power increased from 36% (*β* = 0.04) to 78% (*β* = 0.06, Supplementary Table S3), and then to 98% (*β* = 0.08, Supplementary Table S4). In the pathway analysis, the comparison model KMgene yielded a significantly lower power compared to KMR-LIN-m4 for the same simulation scenario (*N* = 1,000, *m* = 4). This effect increased with increasing effect size β.

### 3.2 Application to the PsyCourse Study

In our real-world data sample, we analyzed 1,518 individuals with at least one TMT-B measurement including 591 “affective,” 533 “psychotic,” and 394 mentally healthy individuals. The mean age at the first measurement point was 41 years [sd: 13.8] 48% of the samples were female (for more details see Supplementary Table S5). At all four measurement points, the psychotic group attained the highest TMT-B score; only at measurement points 1 and 3 were the differences for the psychotic and affective groups significant (see CI of Supplementary Figure S1). The control group demonstrated at each measurement point a significant difference and attained the lowest TMT-B score (Supplementary Figure S1). Previously, we identified a phenotypic outlier, an individual with the highest possible score at each measurement point assessed. Here we focused on the results without the outlier. The results did not change qualitatively when removing the outlier (data not shown).

Thirteen of the 17 tested pathways overlapped at least with one other pathway in at least one gene. The four independent (i.e., pathways not overlapping) were “ion channel transport,” “EPH-ephrin signaling,” “receptor-type tyrosine-protein phosphatases,” and “regulation of MECP2 expression and activity” (Table 2). We did not find any pathways significantly associated with the phenotype TMT-B after multiple testing correction (*p*-value <0.0033 = α_Gao_) for either applied kernel (LIN, NET, and ANET). However, we identified seven pathways in total as achieving a *p*-value <0.05, which are represented in bold in Table 6 with the respective kernel used. For example, the “synaptic adhesion-like molecules” pathway is nominally significant for the main genetic effect for ANET, NET, and LIN. The “signaling by ERBB4” pathway, which poses as the foundation of our simulation, was nominally significant with all three kernels when testing the main genetic effect. For the time-interaction effect, we identified only one pathway, “ion channel transport,” as nominally significant. This pathway has the smallest *p*-value of all pathways (0.0089). To compare the different kernels, we ranked all pathways according to their *p*-values. Table 6 lists the top five pathways for each kernel stratified by the main genetic and time-interaction effects. For both network kernels, we noted a very similar ranking of the top five pathways in the respective genetic effects, whereas for the linear kernel the detected pathways varied between the genetic effect models. Considering the *p*-value ranking, the “synaptic adhesion-like molecules” pathway stood out as the one with smallest *p*-value (rank 1) in all analyses.

**TABLE 6:** Results of the real-data analyses without outlier.

Kernel type	Genetic effect tested	Pathway	*p*-value
Linear kernel (LIN)	Main genetic effect	**Synaptic adhesion-like molecules**	**0.0389**
		NCAM signaling for neurite out-growth	0.0739
		Ion channel transport	0.1029
		Regulation of MECP2 expression activity	0.2101
		MECP2 regulates neuronal receptors and channels	0.2237
Linear kernel (LIN)	Time-interaction effect	**Ion channel transport**	**0.0089**
		MECP2 regulates neuronal receptors and channels	0.2738
		Synaptic adhesion-like molecules	0.3202
		Long-term potentiation	0.3391
		Receptor-type tyrosine-protein phosphatases	0.3579
Network kernel (NET)	Main genetic effect	**Synaptic adhesion-like molecules**	**0.0171**
		**NCAM signaling for neurite-growth**	**0.0472**
		**Signaling by ERBB4**	**0.0496**
		Long-term potentiation	0.0910
		MECP2 regulates neuronal receptors and channels	0.1038
Network kernel (NET)	Time-interaction effect	Synaptic adhesion-like molecules	0.2282
		NCAM1 interactions	0.2551
		Long-term potentiation	0.2943
		Neurexins and neuroligins	0.3341
		Trafficking of AMPA receptors	0.4404
Size-adjusted network kernel (ANET)	Main genetic effect	**Synaptic adhesion-like molecules**	**0.0174**
		**Signaling by ERBB4**	**0.0419**
		NCAM signaling for neurite out-growth	0.0548
		Long-term potentiation	0.0886
		MECP2 regulates neuronal receptors and channels	0.1059
Size-adjusted network kernel (ANET)	Time-interaction effect	Synaptic adhesion-like molecules	0.2429
		NCAM1 interactions	0.2498
		Long-term potentiation	0.2998
		Neurexins and neuroligins	0.3629
		Trafficking of AMPA receptors	0.4866

The five top ranked pathways (according to *p*-value) are listed for each kernel and genetic effect (main genetic and time-interaction effect). Nominal significant (*p*-value <0.05) pathways are printed in bold.

## 4 Discussion

Here we present long-KMR, a topology-based pathway analysis method for longitudinal data, which applies kernel machine regression. The methodological basis of long-KMR is presented. To create long-KMR the connection of KMR and LMM are exploited. In addition, we use the network kernel (Freytag et al., 2013) integrating network information into the model. A simulation study is conducted to assess the performance of long-KMR. The models applied in the simulation study are displayed in Table 1. Different aspects are studied, including the influence of the number of measurement points and varying pathway densities. We modeled and tested a main genetic effect and a time-interaction effect for association, the latter testing the association of a pathway with the trajectory of the phenotype TMT-B. Furthermore, we considered an approach to analyze a joint model containing the main genetic effect and the time-interaction effect in a computationally effective way. Lastly, we applied long-KMR to a cognitive phenotype from the PsyCourse Study (Budde et al., 2018).

### 4.1 Simulation studies

#### 4.1.1 Number of measurements per individual

As expected, the power of long-KMR increases with growing number of measurement points, in particular for the time-interaction effect. This can be traced back to the information that is added to the model at each measurement point, increasing the probability of detecting an effect. Thus, we also identified a larger power loss when analyzing the time-interaction effect with incomplete phenotype data (missing measurements).

#### 4.1.2 Network kernel

The performance of long-KMR improves further when we apply the network kernel instead of the linear kernel, in particular in the single-effect models. We observe that the network kernel has at least the same power as the linear kernel. The power benefit of the network kernel is more pronounced when testing in the presence of smaller genetic effect sizes. For larger genetic effect sizes the power is already extremely high (approx. 98%–99%, Supplementary Table S4), thus the power increase is less noticeable. This power gain is due to the integration of additional pathway information on gene interactions and network topology (Freytag et al., 2013). Here, the topology characteristics of the pathway network play an important role. As the network kernel was developed to exploit the connection of a pathway (Freytag et al., 2013) we studied the influence of the pathway density, identifying a power increase with decreasing pathway density. The higher the density, the more the respective power of network and linear kernel converged. Mathematically, a pathway with many connections (high density) leads to a denser adjacency matrix N, i.e., N contains mainly ‘1’s. Thus, we do not add a lot of specific information when multiplying GA with N (see definition of network kernel). We integrate more noise into the kernel (when N is highly dense) as we sum up the same effects (sum of rows) and only inflate the similarity values artificially (higher range). Thus, we exclude variations and cover potential effects with noise. Consequently, a candidate pathway should preferentially be studied with respect to its characteristics before applying the network kernel when performing long-KMR.

In the joint model including both main and time-interaction effect, the network kernel demonstrated a slightly different performance for different pathway densities. We consider four measurement points only. The network kernel with density 0.2 (lowest density) still has the highest power but only slightly higher when compared to the linear kernel (approx. 72%–73%). The network kernels with densities 0.5 and 0.8 have surprisingly low power compared to the linear kernel. This phenomenon is perhaps due to the simulation of the genetic effect as purely linear effect and enhanced by the application of two different kernel functions in one model. We simulate the genetic effects in a linear fashion and thus we observe the performance of the network kernel in worst-case scenarios. Nevertheless, the network kernel improved long-KMR slightly when the pathway density is not too high. In general, long-KMR is preferable except when testing a very dense pathway. The latter should be acknowledged and considered when interpreting the results of long-KMR under a specific kernel.

It should also be taken into account that a possible misspecification of a pathway, for example, in the form of wrongly described gene connections leads to an inaccurate pathway topology and pathway characteristics, e.g., density. This can lead to power changes in the analysis. Thus, one of the greatest challenges to topology-based pathway analyses remains the possible inaccuracy and perhaps incompleteness of the studied pathways. Here, future work is required to minimize possible misclassifications. In the future, it would be also worthwhile analyzing other pathway characteristics, e.g., the betweenness centrality or diameter of the pathway, and their influence on power of the long-KMR with the network kernel. However, these aspects should also be considered beforehand in one-measurement settings in order to determine any indication of the performance being affected and thus keep the computational costs associated with the analysis of an extensive longitudinal scenario down to an acceptable level. Additionally, more complex simulation models could be considered, including e.g., genetic effect models in which causal SNP effects interact with each other and the causal SNPs vary between main and interaction effects. Here it is expected that these scenarios are even more advantageous for the network kernel. However, this exceeds the scope of this communication.

#### 4.1.3 Comparison of long-KMR with ANCOVA and KMgene

When comparing the different two-measurement models either for the main effect or for the time-interaction effect, long-KMR has the higher power and is the preferred option, in spite of its longer computation time, in particular when using the network kernel. As expected ANCOVA has lower power. Note that the ANCOVA model only uses the second measurement point as dependent variable (Table 1) and loses information regarding the time effect. For the main genetic effect, we even observed that by applying the network kernel compared to the linear kernel, the power loss resulting from the smaller number of measurement points is reduced.

For the four-measurement models the comparison with KMgene (Yan et al., 2015; Yan et al., 2018) on pathway level reveals that our long-KMR has higher power. In addition, the KMgene type I error rates were slightly inflated (Table 3) for the Fisher method. Thus, we used a second *p*-value combination approach according to Stouffer (Larson et al., 2017), yielding even slightly more inflated *p*-values (data not illustrated). Thus, our approach represents the suitable choice when analyzing a whole pathway. KMgene remains a solid approach when analyzing single genes.

### 4.2 Application to the PsyCourse Study

In our application, a total of seven pathways were nominally significant (Table 6). Six of the seven pathways were associated with TMT-B when testing for the main effect. We looked more closely at the pathways with a *p*-value <0.1, i.e., “synaptic adhesion-like molecules,” “signaling by ERBB4,” “long-term potentiation,” and “NCAM signaling for neurite growth.” The first three pathways contain the gene DLG4. This synaptic gene encodes for the density protein 95 (PSD95) and plays a critical role in the activity regulation of NMDA (N-methyl-d-aspartate) receptors in schizophrenic patients (Cheng et al., 2010; Tian et al., 2017). It is important for learning and memory (Tian et al., 2017) and as a predictor of cognitive deficits (Fan et al., 2018). DLG4 is also part of the complex DLG4-NMDA-DLGAP1, which was associated with influencing executive functions, in particular the set-shifting abilities (cognitive flexibility) in attention deficit hyperactivity disorder individuals (Fan et al., 2018). NMDA receptors, which are highly influenced by DLG4, are important in many neuropsychiatric disorders that have a cognitive flexibility impairment (Fan et al., 2018), e.g., schizophrenia (Cheng et al., 2010). Two other schizophrenia susceptibility genes are NRG1 and ERBB4 (Banerjee et al., 2010; Tian et al., 2017), which are part of the “signaling by ERBB4” and “long-term potentiation” pathway together with DLG4. The signaling pathway of NRG1 and ERBB4 has been identified as influencing the transmission of glutamate and GABA (Banerjee et al., 2010), which are implicated in playing a role in executive functions (Hatoum et al., 2020). NRG1-ERBB4 signaling has also been discussed as a target of gene therapy in adults with neurodevelopmental disorders to reduce cognitive impairment, e.g., in executive functions (Shi and Bergson, 2020). They modulate different synaptic processes, such as long-term potentiation, and are essential for the development of the nervous system (Ledonne and Mercuri, 2019), proper brain function and cognitive processes (Ledonne and Mercuri, 2019). The “long-term potentiation” pathway is also strongly influenced by the above-mentioned NMDA glutamate receptors and is strongly involved with learning and memory processes (Lisman et al., 2012; Lüscher and Malenka, 2012). For the fourth pathway, “NCAM signaling for neurite outgrowth,” the neural cell adhesion molecule (NCAM) also plays an important role in the nervous system (Li et al., 2013).

Of the seven pathways nominally significant, “ion channel transport” was the only pathway to prove significant for the time-interaction effect and when modelled with the linear kernel. This pathway had the lowest *p*-value (0.0089). Ion channels are implicated in influencing the susceptibility to or the pathogenesis of psychiatric diseases (Imbrici et al., 2013), and are integral to synaptic functioning.

## Conclusion

Overall, we demonstrated that our longitudinal topology-based pathway analysis displays a power gain and a great flexibility to model pathways and genetic effects. Our approach enables the choice between the popular linear kernel and a network kernel that integrates pathway topology information. The latter demonstrated superiority depending on the density of the pathway of interest. The approach is implemented as the R package kalpra, which is available at https://gitlab.gwdg.de/bernadette.wendel/kalpra.

## Data Availability

The data analyzed in this study is subject to the following licenses/restrictions: Due to the sensitivity of individual genetic information, the dataset presented in this study are available upon reasonable request. Requests to access these datasets should be directed to the authors.
