# Supplementary material for: Kalpra: A kernel approach for longitudinal pathway regression analysis integrating network information with an application to the longitudinal PsyCourse Study
Source: Front Genet. 2022 Dec 6;13:1015885. doi: 10.3389/fgene.2022.1015885 (PMC9767414; doi:10.3389/fgene.2022.1015885)
Supplement: Supplementary file 1 [file DataSheet1.docx]

Supplementary Material

# Supplementary Figures and Tables

## Supplementary Tables

**Supplementary Table 1.** Run times of exemplary analyses

| **Models** | **Kernel** | |
| --- | --- | --- |
|  | Linear Kernel | Network kernel |
| **Simulation (N=1000) – analysis of a single pathway** | | |
| ANCOVA* | 00.55 sec [00.54 sec] | 00.97 sec [00.96 sec] |
| Long-KMR with m=2 | 08.24 sec [07.98 sec] | 15.46 sec [15.18 sec] |
| Long-KMR with m=4 | 35.00 sec [22.86 sec] | 91.01 sec [89.65 sec] |
| KMgene** with m=4 | 08.29 sec [08.05 sec] | - |
| **Real data example (N=1517) – analysis of 17 pathways** | | |
| Baseline (m=1) | 38.31 sec [37.85 sec] | 26.45 sec [26.18 sec] |
| Longitudinal (m=4) | 496.73 sec [488.73 sec] | 861.74 sec [852.98 sec] |

We provide the run time (=time actually needed to finalize the process), which is required to fit the model of the KMR analysis and to compute the p-value. The time in brackets is the CPU time in seconds (sec). For the simulated data, we determine the computation time for an individual pathway consisting of 19 genes with a pathway density of 0.5 (d0.5). The run times displayed for the real-data example provides information for the analysis of 17 pathways.

*The ANCOVA model is applied as pre/post-analysis (two measurements). Due to the characteristics of the ANCOVA model (see Table 1) it uses only one measurement as dependent variable thus it reduces to a KMR with one measurement (baseline).

**For KMgene, we provide the added computation times relating to the analysis of 19 single genes.

Note that the actual runtime can vary as it also depends on the computing power. When applying real-data, the run time is also largely depending data structure, e.g. the number of genotyped SNPs, size of pathway.

**Supplementary Table 2.** Type I error rates of the simulation studies.

| **Models** | **Estimated type I error rate (%)** | | | |
| --- | --- | --- | --- | --- |
|  | α = 5% | α = 1% | α = 0.5% | α = 0.1% |
| KMR-LIN-m2_25MAR | 4.44 | 0.78 | 0.37 | 0.06 |
| KMR-NET-d0.8-m2_25MAR | 4.95 | 0.99 | 0.48 | 0.09 |
| KMR-NET-d0.5-m2_25MAR | 4.93 | 0.99 | 0.46 | 0.07 |
| KMR-NET-d0.2-m2_25MAR | 4.98 | 1.00 | 0.47 | 0.07 |
| KMR-LIN-m4_25MAR | 4.47 | 0.84 | 0.40 | 0.08 |
| KMR-LIN-m4_50MAR | 4.38 | 0.82 | 0.41 | 0.09 |
| KMR-NET-d0.2-m4_25MAR | 4.95 | 1.00 | 0.49 | 0.09 |
| KMR-NET-d0.2-m4_50MAR | 4.93 | 0.98 | 0.48 | 0.09 |
| KMR-NET-d0.5-m4_25MAR | 4.94 | 0.93 | 0.42 | 0.07 |
| KMR-NET-d0.5-m4_50MAR | 4.88 | 0.93 | 0.47 | 0.08 |
| KMR-NET-d0.8-m4_25MAR | 4.88 | 0.95 | 0.48 | 0.10 |
| KMR-NET-d0.8-m4_50MAR | 4.86 | 0.95 | 0.48 | 0.09 |

Simulated type I error for tests at significance levels of α = 5%, 1%, 0.5% and 0.1% are displayed. The simulations are based on 100,000 runs each with 1000 individuals.

**Supplementary Table** **3.** Power results of the simulation study with effect size **0.06**.

|  | **Genetic effect** | | | | | |
| --- | --- | --- | --- | --- | --- | --- |
|  | Main genetic effect | | | Time-interaction effect | | |
| **Models** | Complete | 25MAR | 50MAR | Complete | 25MAR | 50MAR |
| KMR-LIN-ANCOVA | 26.66%  [26.39;26.94] | - | - | 06.13%  [05.98;06.28] | - | - |
| KMR-LIN-m2 | 74.01%  [73.73;74.28] | 59.91%  [59.61;60.22] | - | 51.26%  [50.95;51.58] | 11.33%  [11.13;11.52] | - |
| KMR-NET-d0.8-m2 | 76.45%  [76.18;76.72] | 65.02%  [64.72;65.32] | - | 56.80%  [56.49;87.11] | 39.19%  [38.90;39.50] | - |
| KMR-NET-d0.5-m2 | 79.80%  [79.54;80.05] | 68.74%  [68.45;69.03] | - | 60.28%  [59.97;60.59] | 41.91%  [41.60;42.21] | - |
| KMR-NET-d0.2-m2 | 81.53%  [81.28;81.77] | 70.61%  [70.32;70.89] | - | 62.13%  [61.83;62.44] | 43.42%  [43.11;43.73] | - |
| KMR-LIN-m4 | 77.47%  [77.21;77.73] | 70.63%  [70.35;70.91] | 55.52%  [55.21;55.83] | 92.66%  [92.50;92.83] | 87.35%  [87.14;87.55] | 71.08%  [70.80;71.36] |
| KMR-NET-d0.8-m4 | 79.19%  [79.29;79.79] | 73.74%  [73.46;74.01] | 61.16%  [60.86;61.47] | 92.58%  [92.42;92.74] | 87.89%  [87.68;88.09] | 74.73%  [74.46;75.00] |
| KMR-NET-d0.5-m4 | 82.72%  [82.48;82.96] | 77.32%  [77.06;77.58] | 64.84%  [64.54;65.14] | 94.26%  [94.12;94.41] | 90.40%  [90.21;90.59] | 78.26%  [78.01;78.52] |
| KMR-NET-d0.2-m4 | 84.16%  [83.93;84.40] | 79.09%  [78.83;79.34] | 66.79%  [66.50;67.09] | 95.04%  [94.91;95.18] | 91.40%  [91.22;91.58] | 79.99%  [79.74;80.24] |
| KMgene* | 66.22%  [65.93;66.51] |  |  | - | - | - |

Simulated power to detect an effect of size 0.06 with a test at significance levels of α = 5% is displayed. The simulations are based on 100,000 runs each with 1000 individuals. Power estimates together with 95% confidence interval are presented for genetic main and time-interaction effects. Phenotype data were either complete or with 25/50% of values missing at random (MAR). Model names correspond with Table 1. *For comparability, the single gene-level p-values of KMgene are combined to a pathway p-value using Fisher’s method.

**Supplementary Table 4**. Power results of the simulation study with effect size **0.08**.

|  | **Genetic effect** | | | | | |
| --- | --- | --- | --- | --- | --- | --- |
|  | Main genetic effect | | | Time-interaction effect | | |
| **Models** | Complete | 25MAR | 50MAR | Complete | 25MAR | 50MAR |
| KMR-LIN-ANCOVA | 45.50%  [48.19;48.81] | - | - | 07.54%  [07.37;07.70] | - | - |
| KMR-LIN-m2 | 96.62%  [96.50;96.73] | 89.75%  [89.57;89.94] | - | 82.88%  [82.65;83.12] | 18.23%  [17.99;18.47] | - |
| KMR-NET-d0.8-m2 | 96.17%  [96.05;96.29] | 90.16%  [89.97;90.35] | - | 84.11%  [83.88;84.34] | 64.26%  [63.96;64.56] | - |
| KMR-NET-d0.5-m2 | 97.26%  [97.16;97.36] | 92.34%  [92.17;92.50] | - | 86.86%  [86.65;87.08] | 68.03%  [67.74;68.32] | - |
| KMR-NET-d0.2-m2 | 97.67%  [97.57;97.76] | 93.20%  [93.04;93.36] | - | 88.14%  [87.93;88.34] | 69.85%  [69.56;70.13] | - |
| KMR-LIN-m4 | 97.57%  [97.47;97.66] | 95.30%  [95.17;95.43] | 86.50%  [86.29;86.71] | 99.84%  [99.82;99.87] | 99.40%  [99.35;99.45] | 95.57%  [95.44;95.70] |
| KMR-NET-d0.8-m4 | 97.20%  [97.10;97.30] | 94.93%  [94.80;95.07] | 94.93%  [94.80;95.07] | 99.73%  [99.70;99.76] | 99.14%  [99.08;99.20] | 95.18%  [95.05;95.31] |
| KMR-NET-d0.5-m4 | 98.04%  [97.95;98.22] | 96.29%  [96.17;96.41] | 90.01%  [89.82;90.19] | 99.83%  [99.80;99.85] | 99.43%  [99.39;99.48] | 96.57%  [96.45;96.68] |
| KMR-NET-d0.2-m4 | 98.35%  [98.27;98.43] | 96.86%  [96.75;96.97] | 91.15%  [90.97;91.32] | 99.86%  [99.84;99.89] | 99.54%  [99.50;99.58] | 97.07%  [96.97;97.18] |
| KMgene* | 75.98%  [75.71;76.24] |  |  | **-** | **-** | **-** |

Simulated power to detect an effect of size 0.08 with a test at significance levels of α = 5% is displayed. The simulations are based on 100,000 runs each with 1000 individuals. Power estimates together with 95% confidence interval are presented for genetic main and time-interaction effects. Phenotype data were either complete or with 25/50% of values missing at random (MAR). Model names correspond with Table 1. *For comparability, the single gene-level p-values of KMgene are combined to a pathway p-value using Fisher’s method.

**Supplementary Table 5**. Phenotype information relating to the first measurement point of the PsyCourse Study

| Phenotypes | Diagnostic groups mean (sd) or percentage | | |
| --- | --- | --- | --- |
|  | Affective | Psychotic | Controls |
| Female | 51.4% | 37.7% | 58.6% |
| Age | 44.9 [13.4] | 43.5 [12.0] | 36.8 [15.2] |
| TMT-B | 83.5 [42.3] | 93.1 [42.8] | 58.6 [24.5] |
| **Time effect on lgTMT-B** |  |  |  |
| β [95% CI] | 0.96 [0.95;0.97] | 0.95 [0.94;0.97] | 0.96 [0.95;0.97] |
| p-value | 4.93×10^-11^ | 1.16×10^-13^ | 8.62×10^-15^ |

The mean and standard deviation (sd) of the age at first measurement and the TMT-B for each diagnostic group are provided. The LMM results testing the time effect on lgTMT-B within each diagnostic group are displayed. The effect estimates β of lgTMT-B are transformed back to their original scale.

## Supplementary Figures


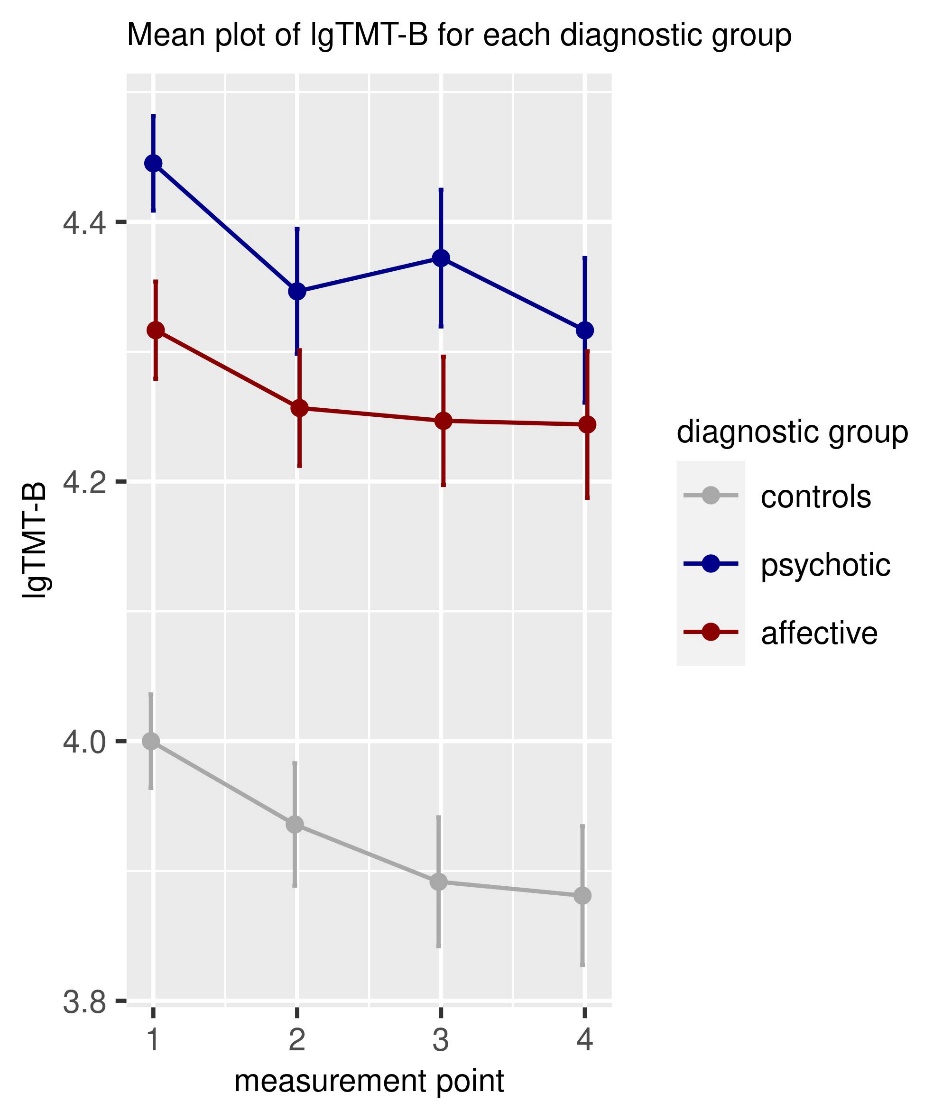


**Supplementary Figure 1.** Longitudinal course of lgTMT-B score (time in seconds) for each diagnostic group (affective, psychotic and controls). Displayed are means with 95% CI for each measurement point 1,2,3,4, approximately 6 months apart.
